# The effects of early neurodevelopmental Bobath approach and mobilization on quadriceps muscle thickness in stroke patients

**DOI:** 10.3906/sag-1808-83

**Published:** 2019-02-11

**Authors:** Arzu GÜÇLÜ GÜNDÜZ, Gökhan YAZICI, Çağla ÖZKUL, Hamit KÜÇÜK, Hale Zeynep BATUR ÇAĞLAYAN, Bijen NAZLIEL

**Affiliations:** 1 Department of Physiotherapy and Rehabilitation, Faculty of Health Sciences, Gazi University, Ankara Turkey; 2 Division of Internal Medicine, Department of Rheumatology, Faculty of Medicine, Gazi University, Ankara Turkey; 3 Department of Neurology, Faculty of Medicine, Gazi University, Ankara Turkey

**Keywords:** Stroke, muscle thickness, neurodevelopmental, Bobath, ultrasonography

## Abstract

**Background/aim:**

Following stroke, damage to the central nervous system and adaptive changes in muscle tissue are factors responsible for the loss of muscle strength. Even though it is suggested that early physiotherapy and mobilization prevent structural adaptive changes in muscle tissue, studies regarding this issue are insufficient. The aim of this study is to investigate the effects of early physiotherapy and mobilization on quadriceps muscle thickness (QMT) in stroke patients.

**Materials and methods:**

Twelve stroke patients who were admitted to the neurology intensive care unit and 13 healthy controls were included in the study. QMT was examined at admission and discharge for each subject. Additionally, functional extremity movements, balance, and functional ambulation status were evaluated with the Stroke Rehabilitation Assessment of Movement Scale (STREAM). All of the patients were mobilized as early as possible by a physiotherapist and included in a treatment program consisting of the neurodevelopmental Bobath approach.

**Results:**

The patients’ QMT values at admission and discharge were found to be similar to those of the healthy control group (P > 0.05). When the patients’ QMT at the time of admission and discharge were compared, it was seen that the affected side and the nonaffected side were similar (P > 0.05). Additionally, when the admission and discharge results were compared, improvements in functional extremity movements, balance, and functional ambulation levels were observed (P < 0.05).

**Conclusion:**

It can be seen that QMT can be preserved and functional improvements can be provided through intense physiotherapy and mobilization initiated in the early period following stroke.

## 1. Introduction

Hemiparesis, which occurs after stroke, is one of the leading reasons for disability and prevents 30% of the patients from walking without aid (1). In stroke cases that result in hemiparesis or hemiplegia, problems in tonus and loss of strength are the most striking complications encountered in muscles.

As the problems caused by tonus decrease in muscle strength arising from the upper motor neuron lesion continue, adaptive structural changes take place in muscles. It has been shown that these changes appear within 4 to 30 h of cerebral infarct (2). These adaptive changes that emerge in the early period after stroke are related to the transsynaptic inhibition of the spinal alpha motor neurons, which innervate muscles, leading to a decrease in the amount of motor units. On the other hand, it would be wrong to associate the cause of the changes occurring in the muscles only with the lesion in the brain. The systematic effects of strokes are usually ignored. However, the decreases in the metabolic and contractile capacity of the muscles and the local inflammatory milieu play important roles in muscle wasting, as well (3). 

The most important structural change that occurs in the muscles after a stroke is the shift between muscle fiber types. Following a stroke, a shift towards fast twitch (myosin heavy chain, MHC type IIa and IIx) isoforms, which are more dependent on anaerobic metabolism, is seen, and the number of slow twitch (mitochondria-rich slow twitch, MHC type I) fibers starts to decrease. This shift in the fiber type is the strongest predictor of functional capacity impairments, such as gait disabilities, which occur after stroke (4).

Inactivity and immobilization that take place after stroke also play a great role in the maladaptive changes occurring in muscles. Inactivity results in insulin resistance and this in turn disrupts the glucose metabolism (5). In a study performed on healthy individuals who had 10 days of bed rest, it was shown that a 30% decrease in muscle protein synthesis, a 6% decrease in fat-free leg mass, and a 16% decrease in muscle strength had occurred (6). A decrease in the cross-sectional area of the muscle fiber and an increase in the intramuscular fat storage appear after strokes in both paretic and nonparetic limbs between the 3rd and 6th weeks (5,7,8). From this point of view, maladaptive changes in muscles are thought to be largely responsible for poststroke disability. According to Scherbakov et al., exercise interventions may be useful in preventing muscle wasting and restoring physical capacity and mobility (9). Additionally, a systematic review and guidelines for adult stroke rehabilitation reported that in stroke patients physical rehabilitation interventions improve functional outcomes and reduce the length of stay in the hospital (10–12). These studies show that functional gains in poststroke rehabilitation are limited; however, the physiological factors causing this limitation have not been proven. Moreover, it is stated that there is a need for studies that put forth the most effective dosage, duration, and intensity of physiotherapy strategies after stroke.

Nozoe et al. analyzed the changes in quadriceps muscle thickness (QMT) with ultrasonography in 16 acute nonambulatory stroke patients with an average age of 72.1 years. In this study, it was shown that QMT decreased in the paretic limb every week during 3 weeks of assessment (13). In another study, Nozoe et al. showed that while the decrease in muscle thickness is related to disease severity and positive CRP on admission, it is not related to nutritional status (14). In both of these studies, the patients were admitted to an early rehabilitation program for 3 weeks and were mobilized within 1 week of being admitted to the hospital. These studies by Nozoe et al. show that, despite early rehabilitation, muscle thickness decreases in both limbs in nonambulatory patients after stroke (13,14).

These are the first studies indicating that adaptive changes begin in muscles in the acute period after a stroke (13,14). On the other hand, our clinical experience shows that muscle wasting after a stroke differs from patient to patient. Factors such as the severity of the stroke, the patient’s ability to access physiotherapy in the early period, and accompanying complications seem to influence this process. Additionally, it is observed that whether exercise and early mobilization have preventive effects on muscle wasting and to what extent they are preventive, as well as which dose, timing, and type of exercise are effective, are among the topics to be addressed.

The neurodevelopmental Bobath approach is a method that involves regulating the muscle tonus and allowing the restoration of functional movement via motor learning principles. This approach adopts an understanding of treatment that is spread throughout the day. However, there are no studies evaluating the efficiency of this approach in the early period of stroke and its effects on muscle thickness. We believe that via the Bobath approach, which emphasizes intensive treatment, muscle thickness can be preserved and the functional level can be improved. Therefore, the aim of this study is to evaluate the effects of the neurodevelopmental Bobath approach on QMT and to compare the QMT of stroke patients with healthy individuals who are of similar age and sex. 

## 2. Materials and methods

### 2.1. Participants and design

Patients who were admitted to the neurology intensive care unit of Gazi University, Ankara, Turkey, with a diagnosis of ischemic stroke were included in the study. The inclusion criteria consisted of a disability level of ≤5 according to the Modified Rankin Scale and a score of 15 according to the Glasgow Coma Scale. The exclusion criteria were being <18 years of age and having recurrent strokes, acute coronary diseases, rheumatologic diseases, or other accompanying neurological diseases. The study was approved by the Gazi University Ethics Committee. 

The neurological examinations of the patients who were brought to Gazi University Hospital with stroke findings were carried out by a neurologist, the stroke diagnosis was confirmed, and their medical treatments were initiated. Suitable patients were referred to the physiotherapy and rehabilitation program. The patients and their caregivers were informed about the study and informed consent was obtained from each patient.

After the physiotherapy evaluation, the patients were evaluated by a radiologist in terms of QMT. In order to compare muscle thickness and to obtain reference values, healthy individuals were included the study and their QMT was evaluated. The control group consisted of sedentary people and, due to the physiological changes that occur with age, they were selected to have ages similar to those of the patients. 

The physiotherapy and mobilization program was initiated after the patients had enough rest on the same day, following the evaluation. The program was applied in the neurology intensive care unit or neurology service.

### 2.2. Evaluation and procedure

For each subject, the age, sex, localization of ischemic stroke, affected side, timing of the first ultrasound imaging, timing of the first mobilization, and duration of hospitalization were recorded. Disease severity, QMT, functional mobility level, disability level, and motor performance were evaluated.

#### 2.2.1. National Institutes of Health Stroke Scale (NIHSS)

The neurological state of the stroke patients and the severity of the stroke were evaluated by the neurologist via the NIHSS. This scale evaluates the consciousness level, sight, visual field, facial symmetry, extremity strength, extremity ataxia, sensory loss, neglect, dysarthria, and aphasia. The total score ranges between 0 and 42. According to this score, the seriousness of the stroke is classified as severe, moderate, or mild. Seven points or below is defined as mild, 8 to 14 points is defined as moderate, and 15 points or more is defined as severe stroke (15).

#### 2.2.2. Oxfordshire classification

According to this scale, individuals were defined to have ischemia or hemorrhage of total anterior circulation, partial anterior circulation, lacunar, or posterior circulation (16). 

#### 2.2.3. Modified Rankin Scale (mRS)

This scale is used to categorize the level of functional independence with reference to prestroke activities. Disability is defined in this scale on 7 levels where 0 points means the patient has a completely normal functional state, 1 point means no disability despite symptoms, 2 points means slight disability, 3 points means moderate disability, 4 points means moderately severe disability, 5 points means severe disability, and 6 points means death (17).

#### 2.2.4. Quadriceps muscle thickness (QMT)

QMT of the musculus rectus femoris (RF), musculus vastus medialis obliques (VMO), musculus vastus lateralis obliques (VLO), and musculus vastus intermedius (VI) were measured using B-mode ultrasound imaging (LOGIQ P5, GE Healthcare Japan, Tokyo, Japan) with a 7–12 MHz linear transducer. All the measurements were performed by the same medical doctor according to the method of Giles et al. (18). During the examination, care was taken to maintain the same standardized supine position. The depth of the image was adjusted until the femur was visible in the center of the screen, and the gain was adjusted until muscle boundaries were visible on screen. Three images were taken of each muscle and saved in an unidentified format for the subsequent analysis and the average of these three measurements was taken. QMT was examined in the paretic and nonparetic limbs during admission and at discharge from the hospital. To locate the measurement sites of the quadriceps muscles, the distance between the superior part of the patella and the anterior superior iliac spine was noted. Using the superior part of the patella as the reference point, the VIM, VLO, and RF were measured at 50% of this distance. To locate the VLO, measurements were taken at 10% of thigh circumference in the lateral direction, and for VMO at 10 cm above the patella (19). 

#### 2.2.6. Stroke Rehabilitation Assessment of Movement Scale (STREAM)

This scale was used for evaluating upper and lower extremity movements (scored on a 3-point ordinal scale, 40 points total) and basic mobility activities (scored on a 4-point ordinal scale, 30 points total) with 30 items (20). The total score is 70. Voluntary movements and basic mobility activities were scored as 0, 1 (a, b, c), 2 and 3 with the ordinal scale. Scores of 1a, 1b, and 1c were scored as 1 point in the statistical analyses. High scores indicate that the motor dysfunction is low, whereas lower scores indicate that the motor dysfunction is high.

#### 2.2.7. Berg Balance Scale (BBS)

The BBS consists of 14 tests that evaluate balance during postural changes in different positions and during movement. The highest score is 56 with this scale (21).

#### 2.2.8. Functional Ambulation Classification (FAC)

This scale was used to determine the level of functional ambulation: 0, the patient cannot walk, or can walk only in parallel bars or with the assistance of two people; 1, the patient can walk with continuous manual contact and monitoring; 2, the patient can walk with the assistance of one person to assist balance without carrying the patient’s weight; 3, the patient can physically walk on level surfaces without manual contact with another person; 4, the patient can walk independently on level surfaces but requires supervision or physical assistance in stairs, inclines, or uneven surfaces; 5, the patient can walk independently with all kinds of speeds and on all types of ground (22).

### 2.3. Intervention

#### 2.3.1. Medical treatment

The patients with acute ischemic stroke were treated with acute reperfusion therapies within the first hours to restore blood flow to ischemic brain tissue. The patients were discharged after completion of diagnostic work-up for ischemic stroke (11).

#### 2.3.2. Neurodevelopmental Bobath approach and mobilization

Bobath exercises aim at developing and regulating normal tonus, preventing abnormal movement patterns, developing a normal sense of movement, developing functional effective movement and increasing its quality, transferring weight to the hemiparetic limb while sitting and standing, developing symmetrical posture, and developing trunk stability and balance (23,24). These exercises were applied in supine, side-lying, sitting, standing, and walking positions. Prior to and after all the sessions, the pulse, respiration frequencies, and blood pressures of all the patients were measured. The patients, their caregivers, and other health personnel were educated on positions that the patients should take during the day. They were instructed on activities they could perform at other times of the day. In cases where the systolic blood pressure was between 120 and 220 mmHg, oxygen saturation was >92% SaO2 (O2 supported or not), heart rate was between 40 and 100 pulses/min, and body heat was <38.5 °C, the treatment was initiated (25). According to their clinical conditions, the patients were seated on the edges of their beds or in wheelchairs or helped in standing up starting from the first session. All of the patients were included in the program twice a day for 60 min for each session and the program was carried out 5 days a week until the patients were discharged.

### 2.4. Statistical analysis

All data were analyzed using SPSS 21 for Windows (IBM Corp., Armonk, NY, USA). Because of the small sample size, nonparametric tests were used and data were expressed as median and interquartile range (25th–75th IQR). QMT values of stroke patients (baseline, discharge) and healthy controls were compared using Kruskal–Wallis analysis of variance. The comparisons were performed using the Mann–Whitney U test or Wilcoxon signed-rank test and statistical significance was set at P < 0.05.

## 3. Results

Forty-seven patients were screened for inclusion in the study. One patient who had a stroke in the past, 5 patients with additional neurological diseases, 1 patient who had a carotid stent applied, 17 patients who did not wish to participate in the study, and 2 patients who wished to quit the study were excluded from the study. Due to technical reasons, ultrasonographic evaluation could not be performed for 9 patients. As a result, the study was completed with 12 patients (5 females and 7 males) and 13 healthy control individuals (10 females and 3 males) (Figure).

**Figure 1:**
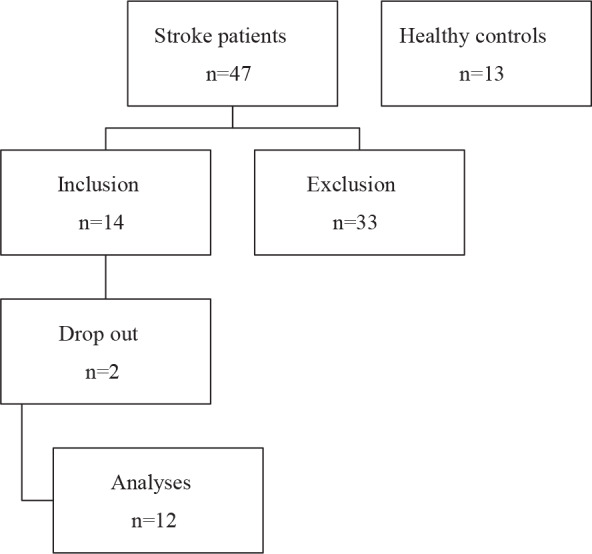
Flow diagram of patients and controls.

The characteristics of the stroke patients and healthy individuals included in the study are shown in Table 1. The severity of the stroke patients according to the NIHSS was an average of 5 (Table 1) and this indicates slight severity. According to the mRS, while the handicap level is slight-medium in 91.7%, it is very severe in 8.3%. According to the FAC, a majority of the patients could walk with the assistance of one or two people (Table 1).

**Table 1:** Characteristics of patients and healthy controls.

		Stroke patients n = 12	Healthy controls n = 13	P
Age (years)		65 (56.5–81.5)	58 (57–66)	0.230
Weight (kg)		77 (70.75–90)	70 (68–78.50)	0.148
Height (cm)		162.5 (156–174.5)	160 (155–166.5)	0.529
Effected side, n (%)				
Right		7 (58.3)	-	-
Left		5 (41.7)	-	-
National Institutes of Health Stroke Scale		5 (4.25–6)	-	-
Modified Rankin Scale, n (%)	2	1 (8.3)		
	3–4	10 (83.4)		
	5	1 (8.3)		
Functional Ambulation Classification (%)	0	8 (66.7)		
	1	1 (8.3)		
	2	1 (8.3)		
	3	2 (16.7)		
Oxfordshire Classification, n (%)	Partial anterior circulation	8 (66.7)		
	Posterior circulation	4 (33.3)		
Duration from stroke onset to hospitalization (min)		60 (30–240)		
Time to initiate physiotherapy and mobilization (h)		79 (55.50–108.50)		
Length of stay in hospital (days)		15 (12–16.75)		
Total rehabilitation session		17 (14–20)		
Time to first QMT measurement (days)		3.27 (2.26–3.27)		
Time to second QMT measurement (days)		16.98 (15.36–20.01)		

P < 0.05 (Mann–Whitney U test). Data are presented as median (IQR) or frequency (%). QMT: Quadriceps muscle thickness.

The stroke patients were individuals who were hospitalized within about 60 min from the beginning of the stroke. The stroke patients were included in the inpatient physiotherapy program within about 79 h after they were admitted to the hospital. The patients stayed at the hospital for an average of 15 days (IQR = 12–16.75) and they were included in the physiotherapy program twice a day for an average of 17 session (IQR = 14–20) in total.

While the initial QMT measurements were carried out on about the 3rd day on average after the stroke, the second QMT measurements were performed on the 17th day after the stroke.

In the first session, while 5 patients could stand and walk, 7 patients were mobilized on the edges of their beds or in wheelchairs. One of the patients who was not able to be mobilized in the first session was mobilized in the 2nd session, one patient was mobilized in the 3rd session, 2 were mobilized in the 4th session, 2 were mobilized in the 5th session, and 1 was mobilized in the 7th session.

QMT muscle thicknesses at baseline and discharge were found to be similar to that of healthy individuals (P > 0.05, Table 2).

**Table 2:** Comparison of quadriceps muscle thickness in stroke patients and healthy controls.

		Affectedmedian (IQR)	Nonaffectedmedian (IQR)	Healthymedian (IQR)	P
Rectus	Initial	2.11 (1.62–2.77)	2 (1.54–2.46)	2.39 (1.97–2.85)	0.296
	Discharge	2.23 (1.92–2.73)	2.26 (1.99–3.20)		0.806
VMO	Initial	1.79 (1.56–2.70)	2 (1.67–2.98)	1.94 (1.62–2.64)	0.851
	Discharge	1.85 (1.57–2.46)	2.19 (1.97–2.50)		0.498
VLO	Initial	2.06 (1.79–2.69)	1.94 (1.65–2.13)	2.27 (1.84–2.60)	0.307
	Discharge	2.19 (1.55–2.28)	2.19 (1.96–2.31)		0.553
VIM	Initial	1.71 (1.36–2.14)	1.86 (1.51–2.39)	1.91 (1.73–2.01)	0.377
	Discharge	1.64 (1.39–2.65)	2.06 (1.49–2.28)		0.624
Total QMT	Initial	7.70 (6.38–10.30)	7.97 (6.68–9.93)	8.93 (7.10–9.92)	0.771
	Discharge	8.04 (7.01–9.82)	8.94 (7.90–10.10)		0.786

P < 0.05 (Kruskal–Wallis analysis of variance). QMT: Quadriceps muscle thickness, Rectus: rectus femoris, VMO: vastus medialis obliques, VLO: vastus lateralis obliques, VIM: vastus intermedius.

When the stroke patients’ affected side and nonaffected side QMT values were compared on the day physiotherapy began, it was seen that there was no difference between the two extremities and that this situation continued to the time of discharge, as well (P > 0.05, Table 3) (except for the initial VIM).

**Table 3:** Comparison of quadriceps muscle thickness before and after early neurodevelopmental Bobath approach in stroke patients.

		Affectedmedian (IQR)	Nonaffectedmedian (IQR)	P^1^
Rectus	Initial	2.11 (1.62–2.77)	2 (1.54–2.46)	0.248
	Discharge	2.23 (1.92–2.73)	2.26 (1.99–3.20)	0.48
	P^2^	0.367	0.38	
VMO	Initial	1.79 (1.56–2.70)	2 (1.67–2.98)	0.367
	Discharge	1.85 (1.57–2.46)	2.19 (1.97–2.50)	0.328
	P^2^	0.583	0.906	
VLO	Initial	2.06 (1.79–2.69)	1.94 (1.65–2.13)	0.248
	Discharge	2.19 (1.55–2.28)	2.19 (1.96–2.31)	0.929
	P^2^	0.79	0.034	
VIM	Initial	1.71 (1.36–2.14)	1.86 (1.51–2.39)	0.031
	Discharge	1.64 (1.39–2.65)	2.06 (1.49–2.28)	0.433
	P^2^	0.367	0.695	
Total	Initial	7.7 (6.38–10.31)	7.97 (6.68–9.93)	0.583
	Discharge	8.04 (7.01–9.82)	8.94 (7.91–10.10)	0.79
	P^2^	0.367	0.239	

P^1^ < 0.05 between affected and nonaffected side (Mann–Whitney U test), P2 < 0.05 within the same extremity (Wilcoxon test). Rectus: Rectus femoris, VMO: vastus medialis obliques, VLO: vastus lateralis obliques, VIM: vastus intermedius.

When the QMT values of the stroke patients were compared before physiotherapy and at the time of discharge, it was seen that there was no difference on the affected side (P > 0.05) and that there was an increase in the VLO muscle thickness on the nonaffected side (P < 0.05, Table 3).

It was also observed that the STREAM, BBS, and FAC scores of stroke patients at the time of discharge had increased (P < 0.05, Table 4).

**Table 4:** Comparison of STREAM, Berg Balance Scale, and Functional Ambulation Classification before and after early neurodevelopmental Bobath approach.

	Initial median (IQR)	Discharge median (IQR)	P
STREAM	37 (18–47.75)	53.5 (45.5–63.75)	0.002
Berg Balance Scale	6 (3–6.75)	32 (21.25–40.75)	0.002
Functional Ambulation Classification	0 (0–1.75)	2.5 (2–3)	0.004

P < 0.05 (Wilcoxon test). STREAM: Stroke Rehabilitation Assessment of Movement Scale.

## 4. Discussion

In this study, the changes that occurred in QMT in the early period following stroke were analyzed. Patients who were hospitalized within an average of 1 h after stroke were included in a rehabilitation session within an average of 79 h. The physiotherapy program consisted of the early and intense neurodevelopmental Bobath approach and mobilization. As a result of the study, it was seen that there was no change in the patients’ QMT (except for the nonaffected side’s VLO) in approximately 15 days. Additionally, functional extremity movements, balance, and ambulation status were improved in patients with stroke. Moreover, when QMT at admission and at the time of discharge were compared in stroke patients and healthy subjects, there was no difference between the groups.

There are two studies that analyzed the change in QMT in the acute stage after stroke (13,14). Sixteen stroke patients with an average age of 72.1 years, whose stroke severity was mild according to the NIHSS in the acute stage and medium or more according to the mRS, were included in the study carried out by Nozoe et al. (13). These patients were mobilized within the first week of their admission to the hospital and were included in a physiotherapy program 5 times a week, each lasting for 40–60 min. In this study, in which mobilization was defined as out-of-bed activities, the mobilization protocol and the type and amount of exercises were not explained in detail. Nozoe et al. analyzed the change in QMT in the first 3 weeks of admission to the hospital and found that QMT in the paretic side decreased every week. It was stated that some change in the nonparetic side in the 2nd week was observed, whereas no change was observed in the 3rd week. As a result, they stated that in nonambulatory stroke survivors, there was a decrease in QMT in particular within the first 2 weeks of admission to the hospital, not only in the paretic but in the nonparetic limb as well (13).

In another study with 31 patients, Nozoe et al. evaluated the changes that took place in QMT within the first 2 weeks of admission to the hospital and similarly showed that there was a decrease in QMT in both extremities (14). When the results of these studies are compared with our results, it can be seen that the measurements of the stroke patients whose ultrasonography evaluations were done on about the 3rd day and the 17th day after the stroke were similar to those in Nozoe et al.’s study. In addition, the severity of the condition and the disability levels of the patients according to the NIHSS and mRS are similar (10,11). However, no change has been observed in our study in any part of QMT with the exception of the nonaffected limb’s VLO. The increase observed in the nonaffected limb’s VLO is considered to be related to transferring more weight to the nonaffected limb. The difference between these two studies and our study may be basically related to the context of the physiotherapy program and mobilization. Unlike Nozoe et al.’s study, the patients were attempted to be mobilized in the soonest time possible and most patients were able to be mobilized in our study (13,14). Nozoe et al. defined mobilization in their study as out-of-bed activities and stated that they were able to mobilize the patients on about the 2nd day. However, it is not clear how much of this involves getting the patients onto their feet (13,14). The quadriceps are activated in particular during weight-transferring activities. Therefore, getting patients onto their feet and transferring weight to the affected extremity in the early period is regarded as one of the most important factors that prevent muscle wasting. 

In addition, a more intense treatment approach was used in our study than in the studies of Nozoe et al. (13,14). The patients were included in the rehabilitation program twice a day and a program consisting of Bobath exercises in supine, side-lying, sitting, and standing positions and during walking was performed. The most important characteristic of the Bobath approach is that it continues throughout the day. Therefore, patients, their caregivers, and other health personnel were educated as to which positions the patients should take during the day and the activities they could perform at other times of the day. In our study, the patients were guided in this manner and given activities according to their functional level. When this aspect of the Bobath approach is considered, it is thought that early period intense physiotherapy is effective in preserving QMT. 

Besides bringing the patient into a standing position and transferring weight in the earliest time possible with intense treatment, the exercises chosen for the applied physiotherapy program are important. For instance, even if it is not possible to get the patients onto their feet, weight transfer to the lower extremities could be stimulated with various exercises. Exercises such as bridge exercises, turning in bed and coming to sitting position, reaching exercises performed in sitting position that transfer weight to the lower extremities, and facilitation methods such as approximations were used. As a result, it is considered that all these approaches are effective in preserving QMT. 

In our study, unlike the study by Nozoe et al. (13), QMT values of stroke patients were compared with those of healthy individuals of similar ages and it was seen that there was no difference in QMT at admittance or discharge. This is considered as another factor that supports the efficiency of the early period neurodevelopmental Bobath approach. 

The results of our study support studies that state that physiotherapy can be effective in terms of muscle wasting, which begins in the early stages of stroke (9,26,27). However, the changes in QMT of stroke patients in the long term have not been analyzed. This is basically related to the unwillingness on the part of the patients to go to the hospital for future evaluations and the difficulties they experience in attending the evaluations at the rehabilitation center where they continue to be treated as inpatients. As a result, studies assessing the effectiveness of physiotherapy programs in the late period of stroke on muscle wasting and studies on whether muscle thickness can be maintained are required.

Another result of our study is that the Bobath approach used in our study caused developments in the patients’ functional extremity movements, balance, and mobility levels at the time of their discharge. With the exception of one individual, all the patients were able to walk with the help or monitoring of one person. Langhamer et al. showed that there was more development in the motor learning program in acute stroke patients (28). The results of this study support the results of our study. The other two studies showed that the Bobath approach applied in the early period develops the patients’ functional levels in individuals with hemorrhagic stroke (29,30). However, more studies are needed to explain the functional benefits of the Bobath approach. Additionally, to obtain the best comparisons, it would be best to have a “no therapy” control group, but we did not have such a control group for ethical reasons. If such a control group were present, we could have seen the effects of exercise and mobilization more clearly. Therefore, due to the absence of such a group we thought that it would be sufficient to take the muscle thickness of healthy individuals as a reference value.

In this study, we assessed only the quadriceps muscle because the quadriceps muscle is one of the major muscles used in standing and walking and we were able to perform limited muscle analysis with ultrasonography. In future studies, the trunk, hip, and ankle muscles, which are key points particularly in postural control, should be analyzed as well.

In conclusion, our study showed that functional performance improves and QMT can be preserved with the neurodevelopmental Bobath approach and early mobilization applied in the early period. The findings of our study give therapists an idea of the type and intensity of treatment to be used for the protection of muscles in the early period of stroke.
